# Full retraction: A.B. El-Remessy, Y. Khalifa, S. Ola, A.S. Ibrahim, G.I. Liou. Cannabidiol protects retinal neurons by preserving glutamine synthetase activity in diabetes. Mol Vis. 2010; 16:1487-1495.

**Published:** 2014-09-08

The authors made substantive errors in figure images of this article such that the hypotheses were not tested and the conclusions were not supported.

On this basis, the Editors formally retract this article from Molecular Vision.

Sincerely,

The Editors of Molecular Vision

